# Hybrid Systems of Oleogels and Probiotic-Loaded Alginate Carriers for Potential Application in Cosmetics

**DOI:** 10.3390/molecules29245984

**Published:** 2024-12-19

**Authors:** Anna Łętocha, Małgorzata Miastkowska, Elżbieta Sikora, Alicja Michalczyk, Marta Liszka-Skoczylas, Mariusz Witczak

**Affiliations:** 1 Cracow University of Technology, Faculty of Chemical Engineering and Technology, 31-155 Cracow, Poland; malgorzata.miastkowska@pk.edu.pl (M.M.); elzbieta.sikora@pk.edu.pl (E.S.); 2 Lukasiewicz—Research Network-Institute of Industrial Organic Chemistry, 03-236 Warsaw, Poland; alicja.michalczyk@ipo.lukasiewicz.gov.pl; 3 Department of Engineering and Machinery for Food Industry, University of Agriculture in Krakow, Balicka Street 122, 30-149 Cracow, Poland; marta.liszka-skoczylas@urk.edu.pl (M.L.-S.); mariusz.witczak@urk.edu.pl (M.W.)

**Keywords:** oleogels, probiotics, alginate microspheres, beads, carriers, hybrid systems, *L. casei*

## Abstract

Oleogels (organogels) are systems resembling a solid substance based on the gelation of organic solvents (oil or non-polar liquid) through components of low molecular weight or oil-soluble polymers. Such compounds are organogelators that produce a thermoreversible three-dimensional gel network that captures liquid organic solvents. Oleogels based on natural oils are attracting more attention due to their numerous advantages, such as their unsaturated fatty acid contents, ease of preparation, and safety of use. As a result of the research, two oleogels were developed, into which freeze-dried alginate carriers with a probiotic, *L. casei*, were incorporated. Two techniques were used to produce probiotic-loaded capsules—extrusion and emulsification. Alginate beads obtained by the extrusion process have a size of approximately 1.2 mm, while much smaller microspheres were obtained using the emulsification technique, ranging in size from 8 to 17 µm. The trehalose was added as a cryoprotectant to improve the survival rate of probiotics in freeze-dried alginate carriers. The encapsulation efficiency for both of the methods applied, the emulsification and the extrusion technique, was high, with levels of 90% and 87%, respectively. The obtained results showed that the production method of probiotic-loaded microspheres influence the bacterial viability. The better strain survival in the developed systems was achieved in the case of microspheres produced by the emulsification (reduction in bacterial cell viability in the range of 1.98–3.97 log in silica oleogel and 2.15–3.81 log in sucragel oleogel after 7 and 30 days of storage) than by the extrusion technique (after a week and a month of oleogel storage, the decrease in cell viability was 2.52–4.52 log in silica oleogel and 2.48–4.44 log in sucragel oleogel).

## 1. Introduction

**Figure 1:**
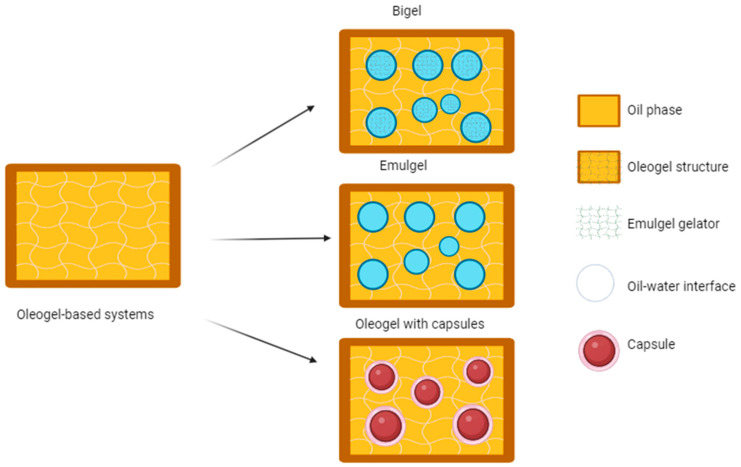
Scheme of oleogel-based systems [by author].

The advantage of oils applied onto the skin is their epidermis-softening properties. They also influence skin hydration and elasticity. In the case of vegetable oils, in addition to their moisturising effect, the skin protective action should be emphasised [1]. Natural oils are characterised by good penetration though the skin; they are incorporated into the lipids of the *stratum corneum* and thus contribute to the improvement of the skin barrier function. The disadvantage of natural oils is their low chemical stability under the influence of light and oxygen. Oils are relatively rarely used as independent carriers of dermatological medicines. Due to their semi-solid consistency, ointments are more convenient to apply [2,3]. A form of medicine or cosmetic formulation that can combine the advantages of ointments and oils are lipophilic gels [1]. Gels are a popular form, in areas such as pharmacy, medicine, the cosmetics industry, tissue engineering, food production, optics, telecommunications, electronics, and polymer plastics processing. There are two main types of gels, hydrogels and organogels, which are distinguished due to the type of liquid phase constituting them [4]. Hydrogels are mainly composed of a hydrophilic polymer network that can absorb large amounts of polar liquid. Oleogels (organogels) are created by stabilising oil in a network created by a gelling medium with a viscous and elastic consistency [5,6,7] (Figure 1). As a result of the heating and cooling of the mixture, interactions occur between the liquid and solid phases [8]. Oil molecules are trapped in a three-dimensional network, which does not cause changes in their chemical composition, and the resulting structure has gel properties [9]. Some types of gels are designed to combine some of the features of hydrogels and oleogels. These hybrid gels, or bigels, are systems that generally contain two immiscible liquid phases that are individually stabilised by independent gelators (Figure 1) [10,11]. Bigels exhibit the advantages of both oil and water phases, including the ability to simultaneously deliver lipophilic and hydrophilic actives and improved viscoelasticity [10,12]. Other hybrid systems are emulgels (a type of hybrid between emulsions and gels) (Figure 1). Emulgels are formed by an emulsification process followed by a gelation process which involves the cross-linking and/or gelation of the compounds present in the mixture.

Two main criteria are used to classify oleogels in the literature. The first is the amount of oleogelator used to obtain the product. As a result, single-component and multi-component (i.e., mixed) organogels are obtained. The second criterion is the type of oleogelator used. As a result, oleogels are formed from low-molecular-weight oleogelators and high-molecular-weight oleogelators [13,14,15,16]. Gelling substances of low molecular weight include, inter alia, edible and vegetable oils compatible with the skin (e.g., olive oil, sweet almond oil) [15,16,17], organic solvents (e.g., benzene, hexane) [18], waxes (beeswax, carnauba wax, candelilla wax) [19,20], colloidal silica [21,22], lecithin [11,23], fatty acids and alcohols, fatty acid esters and/or their mixtures [24,25], ceramides [26], phytosterols [27], and surfactants [11,23]. In turn, high molecular weight oleogelators include polysaccharides (e.g., ethylcellulose), proteins, and others polymers [11,28,29,30].

**Figure 2:**
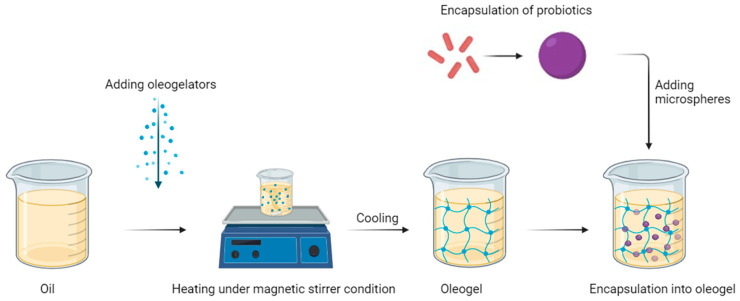
Schematic description of the incorporation of encapsulated probiotics into the oleogel system [by author].

Organogels can be the carriers for encapsulated probiotics as they provide additional protection against unfavourable conditions and reduce the loss of bacterial cells in the hydrocolloid matrix (Figure 1). Probiotics are live microorganisms with biological and physiological effects on the microbiome population, which have the potential to prevent and treat various pathologies, including regulating the microflora by inhibiting pathogenic microorganisms, producing anticancer compounds [31,32] and modulating the immune response [33]. Moreover, the use of a hybrid system and prior encapsulation of probiotics in an additional carrier can enable the increased viability and therapeutic effect of the formulation [34,35] (Figure 2) because in the case of such a hybrid system, there is no need to use a preservative system. The selection of an appropriate shell material is crucial for the stability of the obtained particles. This carrier may be alginate microspheres, which are biocompatible, biodegradable, and non-toxic delivery systems for active substances [36,37].

Thus far, no studies have focused on the application of the oleogel systems as carriers of probiotics encapsulated in hydropolymer capsules. Therefore, the main aim of the work was to find out whether oleogel conditions influenced the survival of bacteria in alginate capsules. For this purpose, stable forms of oleogels were developed to which alginate carriers containing probiotics were incorporated. Trehalose was used as a cryoprotective agent protecting bacterial cells from lyophilisation.

## 2. Results and Discussion

### 2.1. Size and Viability of Bacteria and Encapsulation Efficiency in Microcapsules

Five different types of probiotic-loaded microspheres were developed using two methods, emulsification and extrusion, and trehalose (Thl) was used as a cryoprotectant (Table 1). In addition, two emulsifiers were used to obtain microspheres using the emulsification technique ECO-Tween 80 (Alg/T80) and Span 80 (Alg/S80).

The method used to obtain the probiotic microcapsules influenced their properties. Beads obtained using the extrusion technique have sizes of around 1.2 mm. However, as a result of emulsification, much smaller microspheres were obtained, with sizes ranging from 8 to 17 µm. The obtained results are consistent with the results of other research groups [38,39,40,41], where the use of the extrusion technique resulted in obtaining balls with sizes in the range of millimetres, while emulsification allows obtaining micrometre particles. In the study by Nualkaekul et al. [40] regarding the encapsulation of *L. plantarum* in alginate beads using the extrusion method, capsules with sizes ranging from 2.9 to 3.1 mm were obtained. In the case of the study by Muthukumarasamy et al. [39] regarding the microencapsulation of *L. reuteri* using the extrusion and emulsification methods, the sizes of the beads obtained by extrusion ranged from 2 to 4 mm, while for emulsification, they ranged from 38 to 323 µm. In the study by Mokarram et al. [41], microspheres with probiotics (*L. acidophilus* and *L. rhamnosus*) were obtained using the emulsification technique with sizes of 23.758–75.339 µm.

In the case of cell viability and encapsulation efficiency, the methodology for obtaining the microcapsules did not significantly influence the results. With both emulsification and extrusion (carriers with Tween 80 as an emulsifier in the first technique or with the addition of trehalose), the obtained results were at a similar level and ranged from 9.72 log CFU/g to 9.1 log CFU/g and 90 to 84%, respectively. In the emulsification technique, the use of Span 80 as an emulsifier to produce the W/O pre-emulsion resulted in significantly lower encapsulation efficiency (50%) and a survival rate of *L. casei* of 5.4 log CFU/g. This may be due to the fact that the microspheres obtained in this way had very small sizes of 8.03 µm ± 2.12 µm, and the sizes of the probiotic bacteria themselves ranged from 1 to 4 µm [36,42]. As a result of obtaining such small sizes of microspheres, ineffective encapsulation of the probiotic strain inside the capsule may have occurred, resulting in low encapsulation efficiency.

### 2.2. Survival of Microencapsulated Probiotics During the Freeze-Drying Process

At this stage of the study, the effect of carriers on probiotic survival was analysed. The tests were performed for the capsules obtained by both the emulsification and extrusion techniques and compared to free bacteria not subjected to the encapsulation process.

**Figure 3:**
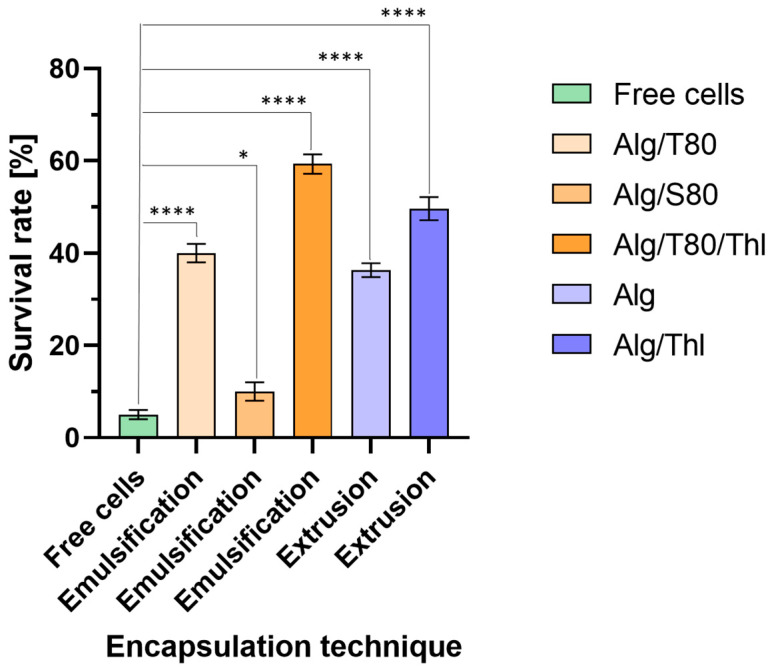
Survival rate of free cells and encapsulated probiotics in the emulsification and extrusion techniques. The bars represent standard deviations of the means according to three independent repeated experiments. *—*p* = 0.05–0.011; ****—*p* ≤ 0.0001.

After the freeze-drying process, the survival rate of encapsulated probiotics was significantly higher than that of free cells (Figure 3).

The lowest survival value of probiotic bacteria was found in the case of freeze-dried Alg/S80 microspheres obtained by emulsification, which is also influenced by the already low initial efficiency of encapsulation—this level was 10% and was similar to the survival rate of freeze-dried, non-encapsulated bacteria (6%). A slightly higher survival rate was observed in the case of Alg/T80 microspheres obtained by emulsification (40%) and Alg capsules obtained by extrusion (35%). In freeze-dried Alg/T80/Thl microspheres obtained by emulsification and Alg/Thl capsules obtained by extrusion, the highest survival rates of the *L. casei* strain were achieved, which were 60% and 50%, respectively.

Reducing the rate of water diffusion from cells leads to the formation of intracellular ice crystals in the freezing phase, which ultimately damages the cell membrane and leads to cell death [43]. However, cells gradually become dehydrated because ice slowly forms outside the cell, which in turn leads to extensive cell damage [44]. The higher survival due to encapsulation is explained by the possible protection of cells against osmotic shock caused by delayed hydration due to the presence of the capsule microenvironment [45,46]. After adding trehalose to the alginate solution as a cryoprotectant, the survival rate of probiotics in microcapsules significantly increased to 20% using the emulsification technique, and 15% in the case of extrusion. As a result of the addition of the cryoprotectant, a strong ion–dipole interaction may occur and hydrogen bonds may be formed between trehalose and cell biomolecules [47,48], which results in the improved survival of the probiotics during freezing and less cell loss in the freezing process.

### 2.3. SEM Analysis

**Figure 4:**
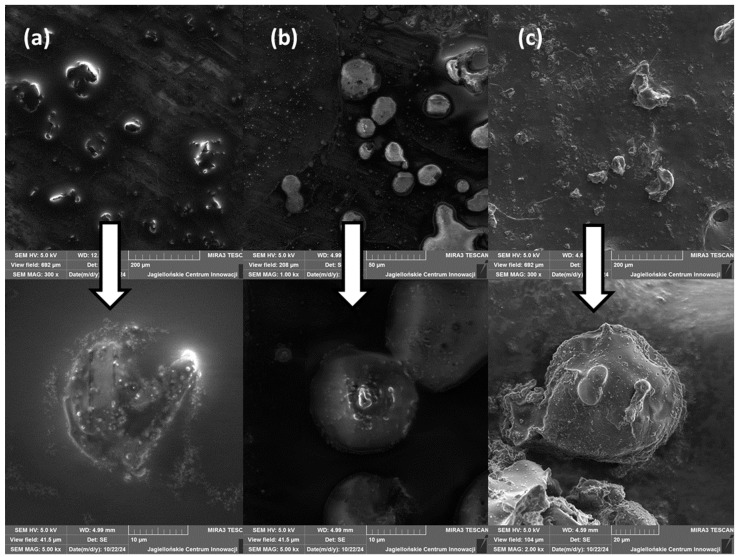
SEM micrographs of freeze-dried microspheres obtained by emulsification: (**a**) alginate–Tween 80 microspheres, (**b**) alginate–Span 80 microspheres, and (**c**) alginate–Tween 80–trehalose microspheres.

**Figure 5:**
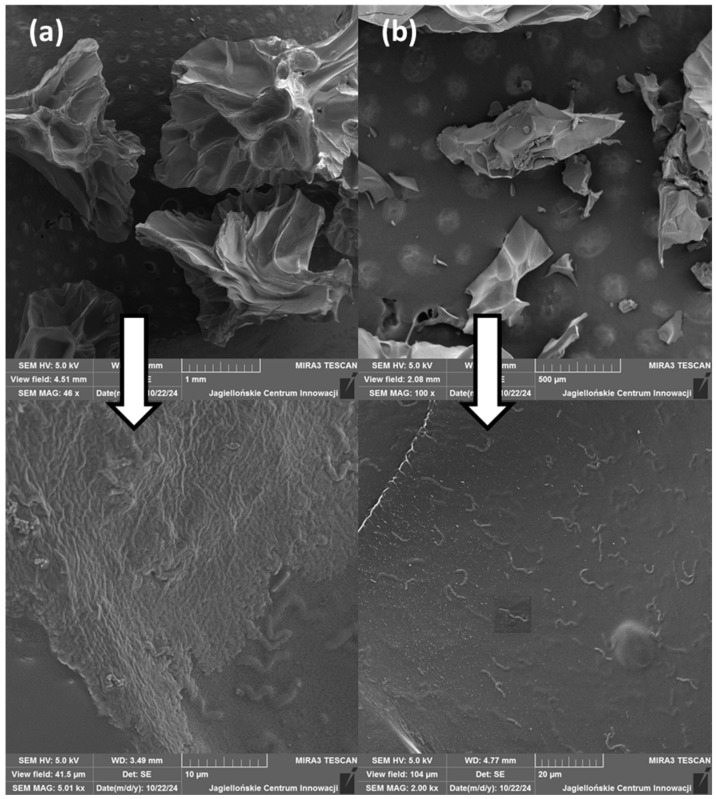
SEM micrographs and cross sections of freeze-dried beads obtained by extrusion: (**a**) alginate beads and (**b**) alginate–trehalose beads.

The surface morphology of the alginate carriers obtained through the emulsification (Figure 4) and extrusion technique were analysed using scanning electron microscopy (SEM) (Figure 5).

The SEM analysis shows that the methods of the probiotic-loaded capsules influence the size and shape of the carriers. SEM images confirm that the particles obtained through the extrusion technique showed significantly larger sizes than the microspheres obtained using the emulsification technique, which is consistent with the literature [38]. Additionally, in the case of the emulsification method, the kind of emulsifier used affects the size of the microspheres. The smallest microspheres were obtained when Span 80 was used as the emulsifying agent, but scanning microscope images showed the lowest visibility of the encapsulated probiotics compared to the larger microspheres obtained when Tween 80 was used.

The use of trehalose as a cryoprotectant, irrespective of the method used, did not affect the size of the obtained particles but influenced their shapes. With the addition of trehalose, more spherical capsules were obtained. Moreover, for the cross section through the beads for the extrusion technique, it can be seen that the addition of trehalose affects the smoothing of the particle structure after lyophilisation. This is consistent with the literature sources, which report that as a result of lyophilisation, the capsules are dehydrated, which can lead to damage of the carrier structures and a change in the shape of the obtained particles [43,44].

### 2.4. Survival of Freeze-Dried Microcapsules in Oleogels

In the next stage of the study concerning probiotic-loaded hybrid systems (freeze-dried capsules incorporated into previously prepared oleogels), the viability of the bacteria was checked.

The initial density of the *L. casei* strain contained in the freeze-dried capsules and incorporated into oleogels was 5.8 log CFU/g. For the emulsification technique, Tween 80 was used as the emulsifier due to its better encapsulation efficiency. A cryoprotectant, trehalose, was also added to the carrier formulation (alginate solution), which had a positive effect on the battery survival rate as a result of the freeze-drying process. Table 2 presents the results of the survival of the probiotic *L. casei* in two oleogel systems.

The bacteria survival rate was assessed immediately after the production of the oleogels, as well as after 7 and 30 days of storage. In the case of oleogels (OG-K and OG-S) containing microspheres with the *L. casei* strain obtained by emulsification, a decrease in the number of probiotic cells by 0.26 log and 0.28 log, respectively, was observed immediately after preparation of the formulation. In studies performed at intervals of 7 and 30 days of organogel storage, a reduction in the viability of bacterial cells was observed in the range of 1.98 to 3.97 log (OG-K) and from 2.15 to 3.81 log (OG-S) compared to the initial value. However, in the case of oleogels containing capsules produced by extrusion, directly after preparing the formulations, the number of *L. casei* cells decreased by 0.32 log (OG-K) and 0.36 log (OG-S). After a week and a month of oleogel storage, the decrease in cell viability was from 2.52 to 4.52 log (OG-K) and from 2.48 to 4.44 log (OG-S).

The literature has so far shown that oleogels can be a carrier for many nutraceuticals while research efforts on the entrapment of probiotics in oleogels are very scarce. Zhuang et al. [49] examined the ability of an oleogel emulsion based on soy lecithin to increase the viability of probiotics. The findings showed that oxidation was significantly slowed down in the soy lecithin-based oleogel emulsion. However, it was not the physical barrier of the oleogel emulsion but the presence of lecithin that increased the viability of probiotics. So far, no positive effect of oleogels on the protection and delivery of probiotics has been demonstrated. Therefore, our study focused on introducing not only probiotics into the formulation but also freeze-dried microcapsules containing probiotics. Based on the studies on the viability of freeze-dried probiotic capsules in oleogels, better strain survival was achieved for microcapsules produced by emulsification (1.83 log in OG-K and 1.99 log in OG-S after 30 days of storage). However, in the case of further research, it would be necessary to focus on increasing the number of cells in the initial freeze-dried microspheres introduced into the product so that their final viability over time would be higher.

### 2.5. Physicochemical Properties of Prepared Oleogels

The physicochemical, microbiological, and rheological studies were performed for oleogels containing optimal microspheres, which had the highest encapsulation efficiency and the best bacteria viability over time (Alg/T80/Thl microspheres).

Two types of stable formulations were prepared. Samples were submitted to an accelerated stability study by centrifugation and to a thermal stability test in order to confirm that the texture of the oleogels had not been altered. The obtained formulations were characterised by pH corresponding to the physiological skin pH. Table 3 describes the physicochemical properties of the prepared oleogels (base formulations and formulations with microspheres).

The results of the mikrocount combi test confirmed the microbiological stability of the formulations. After the incubation time, the results were compared with the evaluation table, which indicates the degree of microbial contamination of the samples [50]. No growth of bacteria, moulds or yeasts was observed.

**Figure 6:**
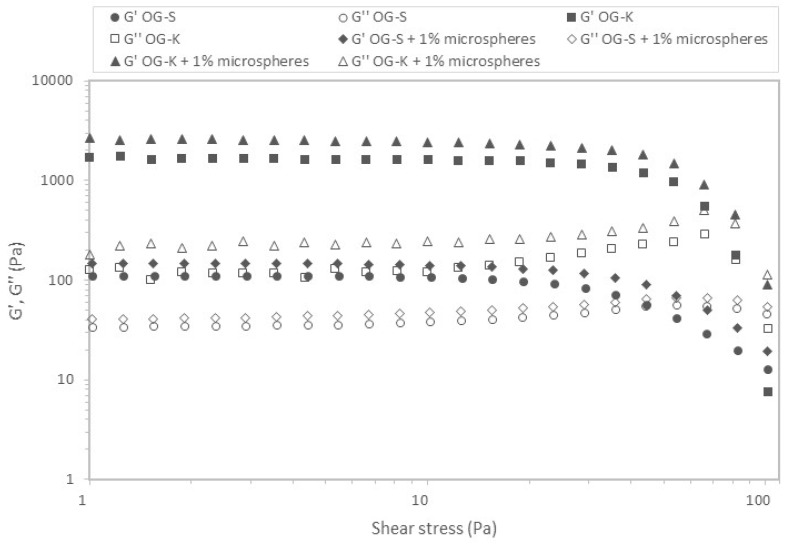
Examples of stress sweeps for the studied oleogels. G′: storage modulus (filled symbols); G″: loss modulus (empty symbols)—temperature 20 °C.

**Figure 7:**
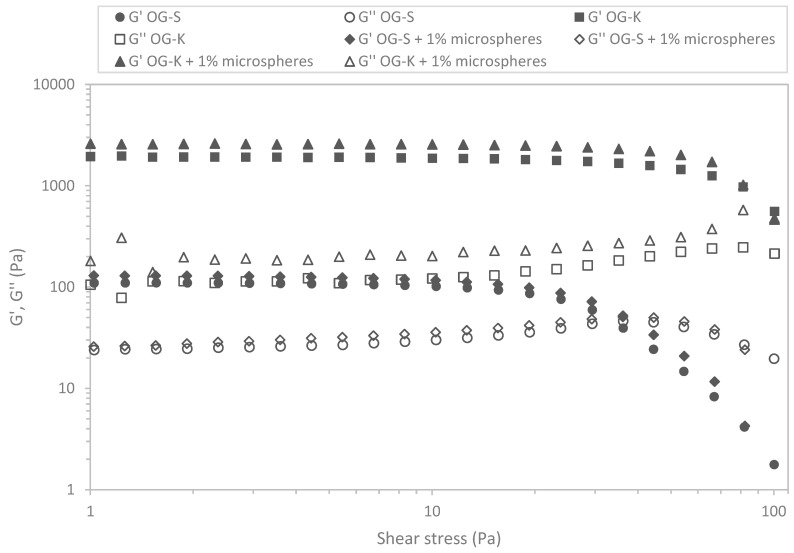
Example stress sweeps for the studied oleogels. G′: storage modulus (filled symbols); G″: loss modulus (empty symbols)—temperature 37 °C.

In the next stage of the research, the viscoelastic properties of the prepared formulations were checked. Figure 6 and Figure 7 show the relation of moduli on stress for the analysed samples.

In all cases, the G′ values were higher than G″, which indicates the predominance of elastic properties over viscous properties. Table 4 and Table 5 list the critical stress values defining the range of linear viscoelasticity and the corresponding stresses.

**Figure 8:**
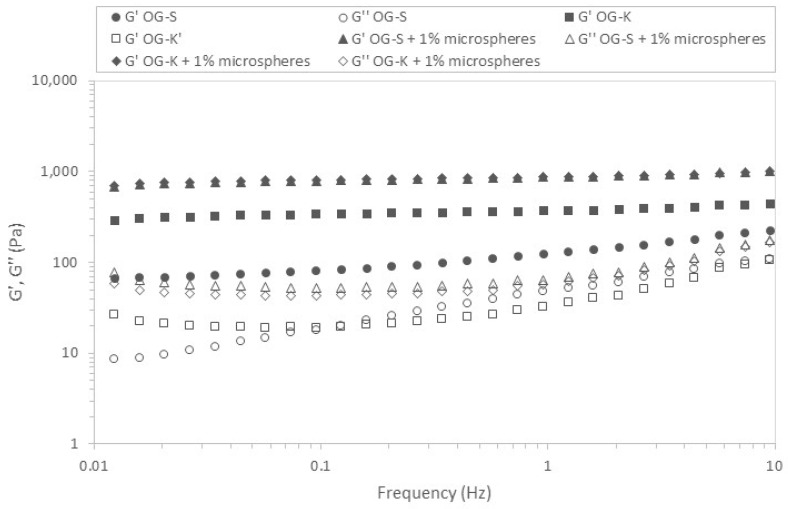
Frequency sweeps for the different oleogels studied. G′: storage modulus (filled symbols); G″: loss modulus (empty symbols)—temperature 20 °C.

**Figure 9:**
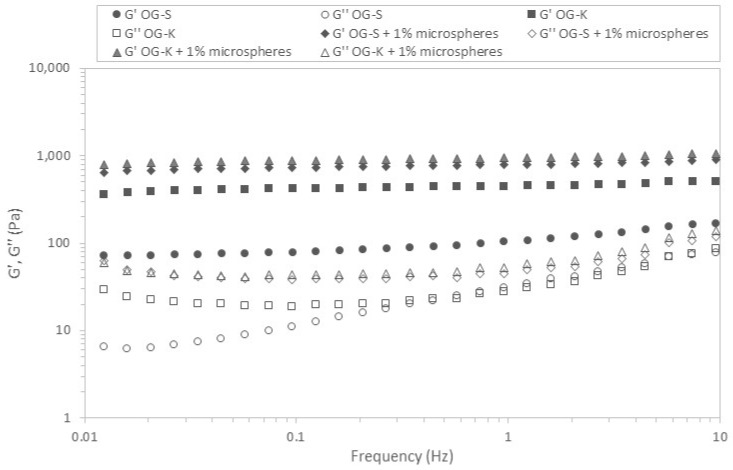
Frequency sweeps for the different oleogels studied. G′: storage modulus (filled symbols); G″: loss modulus (empty symbols)—temperature 37 °C.

The stress values ranged from 10.55 to 35.20 Pa depending on the sample and measurement temperature. Higher values were obtained for samples produced using Aerosil 200 (OG-K), and lower for samples acquired using Sucragel AOF (OG-S). In most cases, the addition of microcapsules caused a decrease in the stress value defining the upper limit of the linear viscoelasticity range; the exception was the sample based on Aerosil 200, for which an increase in this value was found at a higher measurement temperature (37 °C). It should also be emphasised that the OG-K samples (with and without microcapsules) behaved differently, for which an increase in the critical stress value was found with an increase in temperature, while for samples based on Sucragel, a decrease in these values was found. The extension of the linear viscoelasticity range with an increase in temperature can be attributed to the improvement in the stability of the samples with the addition of Aerosil 200 during heating by generating stronger bonds and denser particle packing [21,51]. This is confirmed by the relationships obtained for the mechanical spectra (Figure 8 and Figure 9, Table 4 and Table 5).

The lowest modulus values were obtained for the OG-S base sample, which was also characterised by the highest tan δ value, which indicates a relatively high share of viscous properties. In all analysed cases, the modulus values increased with increasing frequency and the elastic modulus dominated over the loss modulus, which indicates the dominance of elastic over viscous properties. With the exception of the OG-S sample without microcapsules, the remaining samples met the definition of a strong gel (G″/G′ < 0.1) [52]. The addition of microcapsules caused an increase in the modulus values and a decrease in the phase shift angle value in both cases, which indicates an increase in the share of elastic properties and improved stability of the tested samples. In most cases, an increase in temperature caused a decrease in the modulus values and a decrease in the share of viscous properties (decrease in the tan δ value) for samples with the addition of Sucragel AOF. An increase in the storage modulus value with increasing temperature was found for samples produced using Aerosil 200 (in both cases). This rather unusual behaviour of silica samples, as mentioned above, may be related to the sample preparation methodology or lack of treatment at higher temperature, which may lead to significant structural changes during heating, resulting in an increase in the range in which the sample behaves in a stable manner (constant or slight increase in modulus values as a function of stress and extension of the range of linear viscoelasticity). A similar relationship was found in the work of Patel et al. [21], where an increase in gel strength determined by an increase in the G′ modulus value was found after heating. The cited authors observed irreversible restructuring and an increase in the strength of the hydrophilic silica particle network in nonpolar solvents under the influence of heating, which was explained using a fractal gel model. According to the cited work and the work of Wu et al. [51], this is related to the increase in the fractal dimension at higher temperatures as a result of more compact packing and thus stronger hydrogen bonds due to the proximity of the packed particles.

## 3. Materials and Methods

### 3.1. Materials

Alginic acid sodium salt from brown algae, MRS broth and MRS agar (de Man Rogosa Sharpe), and sodium citrate were bought from Sigma Aldrich (Poznań, Poland). The Lactobacillus casei strain ATCC 393 was purchased from American Type Culture Collection (Manassas, VA, USA). ECO-Tween 80 (INCI: Polysorbate 80), Span 80, and Crodamol GTCC (INCI: Caprylic/Capric Triglyceride) were kindly supplied by Croda (Krakow, Poland). Sweet almond oil (INCI: Prunus Amygdalus Dulcis (Sweet Almond) Oil) was bought from Ecospa (Warsaw, Poland), Aerosil 200 (INCI: Silica) was obtained from Evonik Industries AG (Essen, Germany), and Sucragel AOF (INCI: Glycerin, Aqua, Sucrose Laurate) was purchased from Alfa- chemicals (Binfield, UK).

### 3.2. Preparation and Cultivation of Bacteria

The freeze-dried culture of Lactobacillus casei strain ATCC 393 was transferred into MRS broth and incubated for 48 h at 30 °C under aerobic conditions. Harvesting of cells in the log phase was performed by centrifugation at 3000× *g* for 10 min at 4 °C. The cells were washed twice using a 0.9% (*w*/*v*) sterile saline solution. The cell pellets were resuspended in the saline solution and prepared at a final concentration of 10.8 log CFU/g. The numbers of bacteria cells in the suspension were determined by counting them on plates in an MRS agar medium (37 °C, 72 h), using the pour plate inoculation technique.

### 3.3. Preparation of Microcapsules Containing L. casei Bacteria

**Figure 10:**
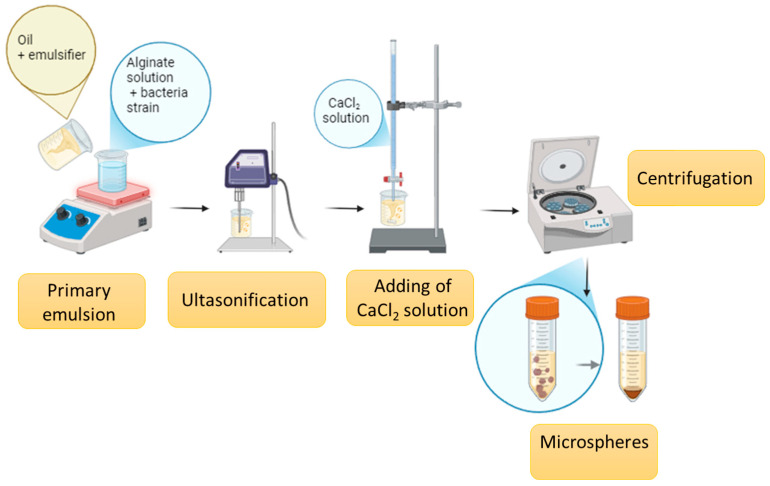
Schematic description of the process of obtaining probiotic microspheres by emulsification [by author].

**Figure 11:**
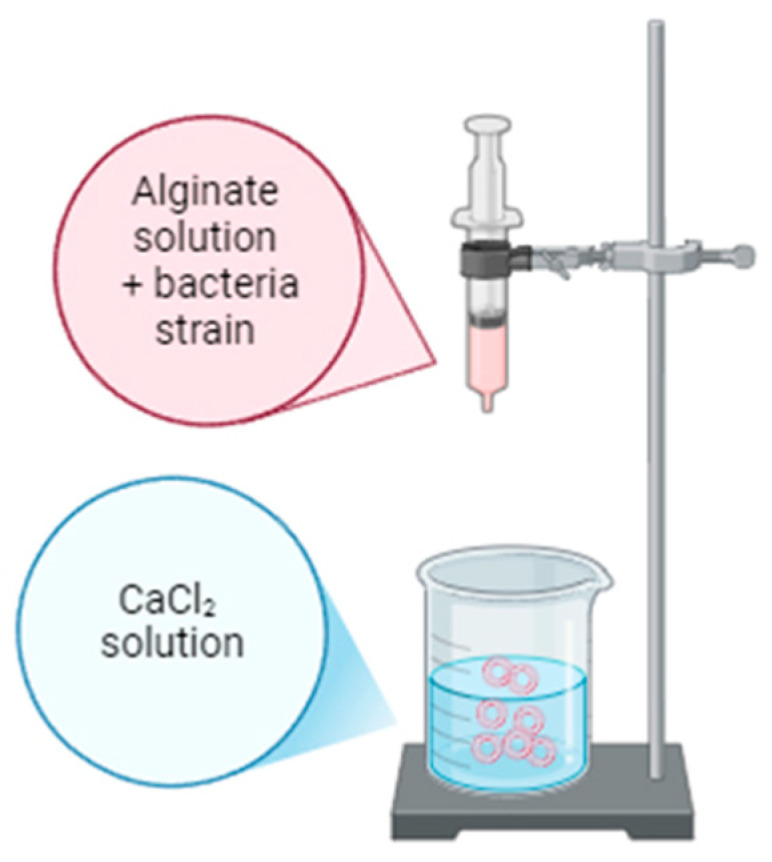
Schematic description of the process of obtaining probiotic microspheres by extrusion [by author].

The encapsulation of probiotics was performed using two techniques. Table 6 shows the types of capsules obtained and a schematic description of the process is shown in Figure 10 and Figure 11.

The first technique, emulsification (Figure 3), was conducted according to the methodology described in our previous article [37] and patent application P. 443812 [53] with slight modification. The initial pre-emulsion consisted of encapsulating material (sodium alginate solution and bacterial suspension) with capric/caprylyl triglycerides, and an emulsifier (ECO-Tween 80 or Span 80) was obtained and then ultrasonicated. Next, CaCl_2_ (1 mol/L) was added in a dropwise manner into a bacteria-loaded pre-emulsion.

For the extrusion technique (Figure 4), the alginate mixtures containing the bacteria strain were added in a dropwise manner into 100 mL of CaCl_2_ (1 mol/L) and left for 30 min for gelation to achieve the alginate beads.

### 3.4. Freeze-Drying of Encapsulated Probiotics

In order to produce dry capsules, they were frozen at −18 °C for 24 h and subsequently dried in a lyophiliser (Lyophilizer Alpha 1-2 LD Plus, Lohra, Germany). As a cryoprotectant, trehalose (10%, *w*/*v*) was added into the alginate solution during the encapsulation procedure in order to improve the survival of the probiotics during the freeze-drying process [54]. Ca-Alg capsules both with and without the cryoprotectant were lyophilised with a freeze drier at −60 °C and 10^−2^ bar for 24 h.

### 3.5. Encapsulation Efficiency and the Viability of Bacteria over Time

To determine the encapsulation efficiency and viability of bacteria, 1 g of the carriers was dissolved in 9 mL of a 0.2 mol L^−1^ sterile sodium citrate solution (pH 6.0) and the entrapped viable bacteria were subsequently counted by the pour plate technique in MRS agar. The encapsulation efficiency (EE) was calculated by Equation (1).

EE = (N/No) × 100%(1)

where the variables were as follows:

N—the number of entrapped viable bacteria cells.

No—the number of free viable bacteria cells before encapsulation.

The viability of probiotic bacteria in the capsules was assessed immediately after the encapsulation process as well as after freeze-drying of the capsules containing the live strain. Moreover, the viability of probiotic bacteria in carriers suspended in the oleogel was assessed immediately after placing them in the gel system, as well as after 7 and 30 days of storage at room temperature. Encapsulation efficiency was also determined for capsules containing the live strain.

### 3.6. Probiotic-Loaded Capsule Morphology Analysis

The morphology of the obtained probiotic carriers was observed using an SEM device—a scanning electron microscope (Mira3-FEG-SEM, Tescan, Brno-Kohoutovice, Czech Republic) with unipolar emission (Schottky emitter) equipped with a cooling table (Peltier) and an energy dispersive X-ray spectrometer EDX (Oxford Instruments). Samples for testing were prepared by quick freezing in liquid nitrogen and then freeze-drying for 24 h [34,37].

### 3.7. Preparation of the Oleogels Containing Encapsulated Probiotic Bacteria

In both cases, probiotic bacteria-loaded capsules obtained both by the emulsification and the extrusion technique were added to the oleogels. For this purpose, capsules containing the addition of trehalose (as the cryoprotective agent) were selected, and Tween 80 was used as the emulsifier in the case of the emulsification method.

#### 3.7.1. Preparation of Organogel with Silica (OG-K)

Table 7 shows the composition of the organogel stabilised by Aerosil 200 (OG-K). Both of the ingredients (almond oil and silica) were mixed using the IKA C-MAG HS7 magnetic mixer at a speed of 500 rpm for 5 min. The process was performed at 25 °C. Finally, 1% of probiotic-loaded capsules were added to the obtained oleogel and the mixture was stirred for 5 min, at the temperature of 25 °C.

#### 3.7.2. Preparation of Organogel with Sucragel (OG-S)

The organogel with sucragel was obtained by weighing the organogelator constituting 30% of the organogel into a beaker and heating it to a temperature of 75 °C (Table 2). Then, using an IKA C-MAG HS7 magnetic stirrer, the organogelator was stirred with the simultaneous slow addition of sweet almond oil at a speed of 500 rpm. The mixture was stirred for 5 min. After the oleogel cooled to 25 °C, 1% of probiotic capsules were added and the mixture was mixed for an additional 5 min.

### 3.8. Study of the Physicochemical Properties of the Oleogels

The study of the physicochemical properties of the prepared oleogels was evaluated using the methodology described in our previous manuscript [55]. The physical stability of the obtained formulations was evaluated using a centrifugation test and a thermal stability test. Briefly, in the centrifugation test, 2 mL samples of each oleogel were submitted to centrifugation at 3500 rpm for the duration of 10 min (EBA 20 Hettich Zentrifugen). In the second method, the formulations were placed in the incubator (+40 °C) for 24 h and refrigerated at −20 °C. The procedure was conducted three times. Next, the formulations were visually inspected for any changes in their texture. The pH values were checked using a Mettler Toledo Seven Easy pH meter equipped with a glass Inlab 410 electrode.

### 3.9. Study of the Microbiological Stability

The microbiological stability of the oleogels were tested using a mikrocount^®^ duo test (Schulke, Norderstedt, Germany). The plastic slides were coated on one side with TTC agar (the medium for bacterial count) and on the other side with Rose Bengal CAF agar (the medium for yeast and mould detection) and were dipped in the product samples for 10 s. The slides were then placed back in the sterile tube and incubated at 30 °C for 48 h. For the detection of yeasts, the incubation time was extended for a further 48 h at 30 °C [50].

### 3.10. Viscoelastic Properties of Base Oleogels and Oleogels with Encapsulated Probiotics

The viscoelastic properties were identified using an oscillation rheometer RS 6000 (Thermo-Haake, Dreieich, Germany). A parallel notched plate system (diameter 35 mm, gap size 1 mm) was used as the measuring element, which enabled the elimination of the slip phenomenon. The prepared samples were placed in the rheometer measuring system and left for 5 min to relax stresses and stabilise the temperature. The tests were performed at two temperatures: 20 °C and 37 °C.

Stress sweep tests were performed in a range from 1 to 100 Pa at a constant frequency of 1 Hz.

The linear viscoelasticity range (LVR) was identified by determining (Haake Rheowin Data Manager 4.93) the point at which the elastic modulus (G′) deviates by 5% from the plateau [56]. The dynamic plastic stress (τ) and the corresponding strain (γ) were determined. Mechanical spectra in the range of 0.01 to 10 Hz were identified in the linear viscoelastic range at constant strain (0.4%). The experimental data are described by power Equations (2) and (3):

(2) $$G^{\prime }=G_{1}^{\prime }\cdot \nu^{m^{\prime }}$$

(3) $$G^{\prime \prime }=G_{1}^{\prime \prime }\cdot \nu^{m^{\prime \prime }}$$

where G′ is the storage modulus (Pa), G″ is the loss modulus (Pa), ν is the frequency (Hz), and G1′, G1″, m′, and m″ are constants.

## 4. Conclusions

In this study, stable hybrid systems were developed, specifically oleogel formulations (OG-K and OG-S) containing freeze-dried capsules with the probiotic *L. casei*. The addition of trehalose (a cryoprotective agent) to the capsule composition improved the bacteria survival rate and influenced the shape of the carriers resulting in more compact and regular microcapsules in the form of freeze-dried powders. Moreover, the wall system (Ca–Alg) protects the probiotics from thermal conditions. SEM analysis confirmed significantly smaller sizes of microspheres obtained in the case of the emulsification technique compared to the spheres obtained by the extrusion method. The use of trehalose did not affect the size of the obtained particles for either method but contributed to the formation of more compact structures.

Studies on the survival of freeze-dried probiotic capsules in oleogels (prepared by both extrusion and emulsification) showed better strain survival in the case of microcapsules prepared using the emulsification technique. In both oleogel systems (OG-K and OG-S), the survival rate of the probiotic strain encapsulated in microspheres was similar. Although the approach based on the introduction of probiotic microcapsules into oleogels is promising, more studies on the delivery of probiotics using such systems should be conducted to verify the advantages of the proposed solution.

## Figures and Tables

**Table 1:** Size and EE of the capsules obtained using the emulsification and extrusion techniques.

Technique	Type of Capsule	Size [µm]	Cell Viability [log CFU g^−1^]	EE [%]
Emulsification	Alg/T80	16.8 ± 3.02	9.61	89
	Alg/S80	8.03 ± 2.12	5.4	50
	Alg/T80/Thl	15.9 ± 4.18	9.72	90
Extrusion	Alg	1216 ± 302.86	9.1	84
	Alg/Thl	1169 ± 412.11	9.4	87

**Table 2:** Viability of encapsulated probiotic bacteria in oleogels.

Oleogel Type	Encapsulation Technique	Type of Microcapsules	Immediately		After 7 Days		After 30 Days	
			Cell Viability [log CFU g^−1^]	Log Reduction	Cell Viability [log CFU g^−1^]	Log Reduction	Cell Viability [log CFU g^−1^]	Log Reduction
OG-K	Emulsification	Alg/T80/Thl	5.54	0.26	3.82	1.98	1.83	3.97
	Extrusion	Alg/Thl	5.48	0.32	3.28	2.52	1.28	4.52
OG-S	Emulsification	Alg/T80/Thl	5.52	0.28	3.65	2.15	1.99	3.81
	Extrusion	Alg/Thl	5.44	0.36	3.32	2.48	1.36	4.44

**Table 3:** Physicochemical properties of oleogels.

	OG-K	OG-K + 1% Microspheres	OG-S	OG-S + 1% Microspheres
**Centrifugation test**	+	+	+	+
**Thermal stability test**	+	+	+	+
**pH**	6.04 (±0.02)	5.92 (±0.03)	6.08 (±0.03)	5.90 (±0.02)

**Table 4:** Mean values of oscillatory tests parameters in oleogels. Amplitude sweeps: end of linear viscoelastic region, LVR (τ and γ). Frequency sweeps: power law parameter fits (G1′, G1″, m′, and m″) and loss tangent at 1 rad/s—temperature 20 °C.

Oleogel	LVR		Frequency Sweeps				
	τ (Pa)	γ	G_1_′ (Pa)	m′	G_1_″ (Pa)	m″	tan δ (1 rad/s)
OG-S	17.73 ± 1.16 ^a^	0.168 ± 0.010 ^d^	131.7 ± 4.4 ^a^	0.209 ± 0.003 ^c^	47.8 ± 0.7 ^b^	0.386 ± 0.009 ^c^	0.271 ± 0.009 ^d^
OG-K	27.54 ± 1.52 ^b^	0.020 ± 0.002 ^b^	376.7 ± 19.1 ^b^	0.052 ± 0.000 ^b^	40.1 ± 1.4 ^a^	0.390 ± 0.010 ^c^	0.059 ± 0.001 ^b^
OG-S + 1% microspheres	16.95 ± 0.71 ^a^	0.120 ± 0.003 ^c^	879.1 ± 40.3 ^c^	0.048 ± 0.002 ^b^	85.4 ± 3.4 ^d^	0.213 ± 0.005 ^a^	0.067 ± 0.001 ^c^
OG-K + 1% microspheres	25.63 ± 0.44 ^b^	0.012 ± 0.000 ^a^	886.2 ± 23.6 ^c^	0.042 ± 0.002 ^a^	74.1 ± 1.5 ^c^	0.265 ± 0.004 ^b^	0.054 ± 0.000 ^a^

OG-S: oleogels with sucragel; OG-K: oleogels with silica. Different letters in each column indicate significance between the means (<0.05) according to Tukey’s test.

**Table 5:** Mean values of oscillatory tests parameters in oleogels. Amplitude sweeps: end of linear viscoelastic region, LVR (τ and γ). Frequency sweeps: power law parameter fits (G1′, G1″, m′, and m″) and loss tangent at 1 rad/s—temperature 37 °C.

Oleogel	LVR		Frequency Sweeps				
	τ (Pa)	γ	G_1_′ (Pa)	m′	G_1_″ (Pa)	m″	tan δ (1 rad/s)
OG-S	12.57 ± 0.56 ^a^	0.124 ± 0.003 ^d^	113.8 ± 4.6 ^a^	0.149 ± 0.006 ^b^	31.6 ± 1.3 ^a^	0.421 ± 0.007 ^d^	0.172 ± 0.002 ^b^
OG-K	30.93 ± 0.37 ^b^	0.021 ± 0.002 ^b^	461.5 ± 20.8 ^b^	0.042 ± 0.002 ^a^	36.2 ± 1.7 ^a^	0.313 ± 0.005 ^c^	0.046 ± 0.001 ^a^
OG-S + 1% microspheres	10.55 ± 0.40 ^a^	0.082 ± 0.003 ^c^	796.7 ± 32.8 ^c^	0.040 ± 0.002 ^a^	60.5 ± 2.5 ^b^	0.179 ± 0.014 ^a^	0.053 ± 0.004 ^a^
OG-K + 1% microspheres	35.20 ± 0.69 ^c^	0.014 ± 0.001 ^a^	952.3 ± 76.8 ^d^	0.036 ± 0.001 ^a^	68.7 ± 7.2 ^b^	0.218 ± 0.018 ^b^	0.049 ± 0.005 ^a^

OG-S: oleogels with sucragel; OG-K: oleogels with silica. Different letters in each column indicate significance between the means (<0.05) according to Tukey’s test.

**Table 6:** The types of capsules obtained with the emulsification and extrusion techniques.

Technique	Type of Capsules	Type of Capsules
**Emulsification**	Alginate + ECO-Tween 80	Alg/T80
	Alginate + Span 80	Alg/S80
	Alginate + ECO-Tween 80 + trehalose	Alg/T80/Thl
**Extrusion**	Alginate	Alg
	Alginate + trehalose	Alg/Thl

**Table 7:** Composition of prepared base oleogels.

Sample No.	Ingredients (Trade Name)	INCI Name	Concentration [%]
OG-K	Aerosil 200	Silica	5
	Sweet almond oil	Prunus Amygdalus Dulcis (Sweet Almond) Oil	95
OG-S	Sucragel AOF	Glycerin, Aqua, Sucrose Laurate	30
	Sweet almond oil	Prunus Amygdalus Dulcis (Sweet Almond) Oil	70

## Data Availability

Data are contained within the article.

## References

[1] Sikorska K., Szulc J., Pietkiewicz J., Sznitowska M. (2009). Oleożele z Kwasem Salicylowym w Praktyce Leku Recepturowego. Polish Pharm..

[2] Marzec A. (2001). Chemia Kosmetyków—Surowce, Półprodukty, Preparatyka Wyrobów.

[3] Placek W. (2005). Rola Podłoży i Emolientów w Profilaktyce i Przywracaniu Bariery Naskórkowej. Dermatol. Estet..

[4] Sowa-Kasprzak K., Zwawiak J., Zaprutko L. (2018). Organogels as Modern Drug Carriers. Polimery.

[5] Żbikowska A., Kupiec M., Onacik-Gür S. (2017). Wpływ Karagenu Na Teksturę I Stabilność Oleożeli Hydroksypropylometylocelulozowych. Acta Agroph..

[6] Co E.D., Marangoni A.G. (2012). Organogels: An Alternative Edible Oil-Structuring Method. JAOCS J. Am. Oil Chem. Soc..

[7] Patel A.R., Dewettinck K. (2015). Comparative Evaluation of Structured Oil Systems: Shellac Oleogel, HPMC Oleogel, and HIPE Gel. Eur. J. Lipid Sci. Technol..

[8] Öğütcü M., Arifoğlu N., Yilmaz E. (2015). Preparation and Characterization of Virgin Olive Oil-Beeswax Oleogel Emulsion Products. JAOCS J. Am. Oil Chem. Soc..

[9] Jang A., Bae W., Hwang H.S., Lee H.G., Lee S. (2015). Evaluation of Canola Oil Oleogels with Candelilla Wax as an Alternative to Shortening in Baked Goods. Food Chem..

[10] Pinto T.C., Martins A.J., Pastrana L., Pereira M.C., Cerqueira M.A. (2021). Oleogel-Based Systems for the Delivery of Bioactive Compounds in Foods. Gels.

[11] Davidovich-Pinhas M., Pal K., Banerjee I. (2018). Oleogels. Polymeric Gels.

[12] Martins A.J., Silva P., Maciel F., Pastrana L.M., Cunha R.L., Cerqueira M.A., Vicente A.A. (2019). Hybrid Gels: Influence of Oleogel/Hydrogel Ratio on Rheological and Textural Properties. Food Res. Int..

[13] Kulawik-pióro A., Miastkowska M. (2021). Polymeric Gels and Their Application in the Treatment of Psoriasis Vulgaris: A Review. Int. J. Mol. Sci..

[14] Shakeel A., Farooq U., Iqbal T., Yasin S., Lupi F.R., Gabriele D. (2019). Key Characteristics and Modelling of Bigels Systems: A Review. Mater. Sci. Eng. C.

[15] Lupi F.R., Gabriele D., Facciolo D., Baldino N., Seta L., de Cindio B. (2012). Effect of Organogelator and Fat Source on Rheological Properties of Olive Oil-Based Organogels. Food Res. Int..

[16] Almeida I.F., Bahia M.F. (2006). Evaluation of the Physical Stability of Two Oleogels. Int. J. Pharm..

[17] Da Pieve S., Calligaris S., Panozzo A., Arrighetti G., Nicoli M.C. (2011). Effect of Monoglyceride Organogel Structure on Cod Liver Oil Stability. Food Res. Int..

[18] Hughes N.E., Marangoni A.G., Wright A.J., Rogers M.A., Rush J.W.E. (2009). Potential Food Applications of Edible Oil Organogels. Trends Food Sci. Technol..

[19] Rocha J.C.B., Lopes J.D., Mascarenhas M.C.N., Arellano D.B., Guerreiro L.M.R., da Cunha R.L. (2013). Thermal and Rheological Properties of Organogels Formed by Sugarcane or Candelilla Wax in Soybean Oil. Food Res. Int..

[20] Toro-Vazquez J.F., Morales-Rueda J.A., Dibildox-Alvarado E., Charó-Alonso M., Alonzo-Macias M., González-Chávez M.M. (2007). Thermal and Textural Properties of Organogels Developed by Candelilla Wax in Safflower Oil. JAOCS J. Am. Oil Chem. Soc..

[21] Patel A.R., Mankoč B., Bin Sintang M.D., Lesaffer A., Dewettinck K. (2015). Fumed Silica-Based Organogels and “aqueous-Organic” Bigels. RSC Adv..

[22] Lu Z., Fassihi R. (2015). Influence of Colloidal Silicon Dioxide on Gel Strength, Robustness, and Adhesive Properties of Diclofenac Gel Formulation for Topical Application. AAPS PharmSciTech.

[23] Shakeel A., Farooq U., Gabriele D., Marangoni A.G., Lupi F.R. (2021). Bigels and Multi-Component Organogels: An Overview from Rheological Perspective. Food Hydrocoll..

[24] Blach C., Gravelle A.J., Peyronel F., Weiss J., Barbut S., Marangoni A.G. (2016). Revisiting the Crystallization Behavior of Stearyl Alcohol: Stearic Acid (SO: SA) Mixtures in Edible Oil. RSC Adv..

[25] Uvanesh K., Sagiri S.S., Senthilguru K., Pramanik K., Banerjee I., Anis A., Al-Zahrani S.M., Pal K. (2016). Effect of Span 60 on the Microstructure, Crystallization Kinetics, and Mechanical Properties of Stearic Acid Oleogels: An In-Depth Analysis. J. Food Sci..

[26] Bin Sintang M.D., Danthine S., Brown A., Van de Walle D., Patel A.R., Tavernier I., Rimaux T., Dewettinck K. (2017). Phytosterols-Induced Viscoelasticity of Oleogels Prepared by Using Monoglycerides. Food Res. Int..

[27] Bot A., Agterof W.G.M. (2006). Structuring of Edible Oils by Mixtures of γ-Oryzanol with β-Sitosterol or Related Phytosterols. JAOCS J. Am. Oil Chem. Soc..

[28] Patel A.R., Cludts N., Sintang M.D.B., Lesaffer A., Dewettinck K. (2014). Edible Oleogels Based on Water Soluble Food Polymers: Preparation, Characterization and Potential Application. Food Funct..

[29] de Vries A., Wesseling A., van der Linden E., Scholten E. (2017). Protein Oleogels from Heat-Set Whey Protein Aggregates. J. Colloid Interface Sci..

[30] Suzuki M., Setoguchi C., Shirai H., Hanabusa K. (2007). Organogelation by Polymer Organogelators with a L-Lysine Derivative: Formation of a Three-Dimensional Network Consisting of Supramolecular and Conventional Polymers. Chem.-A Eur. J..

[31] Zhao W., Wei Z., Xue C. (2022). Recent Advances on Food-Grade Oleogels: Fabrication, Application and Research Trends. Crit. Rev. Food Sci. Nutr..

[32] Legesse Bedada T., Feto T.K., Awoke K.S., Garedew A.D., Yifat F.T., Birri D.J. (2020). Probiotics for Cancer Alternative Prevention and Treatment. Biomed. Pharmacother..

[33] Górska A., Przystupski D., Niemczura M.J., Kulbacka J. (2019). Probiotic Bacteria: A Promising Tool in Cancer Prevention and Therapy. Curr. Microbiol..

[34] Łętocha A., Michalczyk A., Miastkowska M., Sikora E. (2024). Effect of Encapsulation of Lactobacillus Casei in Alginate-Tapioca Flour Microspheres Coated with Different Biopolymers on the Viability of Probiotic Bacteria. ACS Appl. Mater. Interfaces.

[35] Łętocha A., Michalczyk A., Ostrowska P., Miastkowska M., Sikora E. (2024). Probiotics-Loaded Microspheres for Cosmetic Applications. Appl. Sci..

[36] Łętocha A., Miastkowska M., Sikora E. (2022). Preparation and Characteristics of Alginate Microparticles for Food, Pharmaceutical and Cosmetic Applications. Polymers.

[37] Łętocha A., Michalczyk A., Miastkowska M., Sikora E. (2023). Design of Alginate Microspheres Formulation as a Probiotics Carrier. Chem. Process Eng. New Front..

[38] Pupa P., Apiwatsiri P., Sirichokchatchawan W., Pirarat N., Muangsin N., Shah A.A., Prapasarakul N. (2021). The Efficacy of Three Double-Microencapsulation Methods for Preservation of Probiotic Bacteria. Sci. Rep..

[39] Muthukumarasamy P., Holley R.A. (2006). Microbiological and Sensory Quality of Dry Fermented Sausages Containing Alginate-Microencapsulated Lactobacillus Reuteri. Int. J. Food Microbiol..

[40] Nualkaekul S., Lenton D., Cook M.T., Khutoryanskiy V.V., Charalampopoulos D. (2012). Chitosan Coated Alginate Beads for the Survival of Microencapsulated Lactobacillus Plantarum in Pomegranate Juice. Carbohydr. Polym..

[41] Mokarram R.R., Mortazavi S.A., Najafi M.B.H., Shahidi F. (2009). The Influence of Multi Stage Alginate Coating on Survivability of Potential Probiotic Bacteria in Simulated Gastric and Intestinal Juice. Food Res. Int..

[42] Rokka S., Rantamäki P. (2010). Protecting Probiotic Bacteria by Microencapsulation: Challenges for Industrial Applications. Eur. Food Res. Technol..

[43] Halim M., Mohd Mustafa N.A., Othman M., Wasoh H., Kapri M.R., Ariff A.B. (2017). Effect of Encapsulant and Cryoprotectant on the Viability of Probiotic Pediococcus Acidilactici ATCC 8042 during Freeze-Drying and Exposure to High Acidity, Bile Salts and Heat. LWT.

[44] Heidebach T., Först P., Kulozik U. (2010). Influence of Casein-Based Microencapsulation on Freeze-Drying and Storage of Probiotic Cells. J. Food Eng..

[45] Meng X.C., Stanton C., Fitzgerald G.F., Daly C., Ross R.P. (2008). Anhydrobiotics: The Challenges of Drying Probiotic Cultures. Food Chem..

[46] Carvalho A., Teixeira P., Gibbs P., Carvalho A.S., Silva J., Ho P., Teixeira P., Malcata F.X., Gibbs P. (2004). Relevant Factors for the Preparation of Freeze-Dried Lactic Acid Bacteria. Int. Dairy J..

[47] Han C., Xiao Y., Liu E., Su Z., Meng X., Liu B. (2020). Preparation of Ca-Alginate-Whey Protein Isolate Microcapsules for Protection and Delivery of *L. Bulgaricus* and *L. Paracasei*. Int. J. Biol. Macromol..

[48] Fowler A., Toner M. (2006). Cryo-Injury and Biopreservation. Ann. N. Y. Acad. Sci..

[49] Zhuang X., Gaudino N., Clark S., Acevedo N.C. (2021). Novel Lecithin-Based Oleogels and Oleogel Emulsions Delay Lipid Oxidation and Extend Probiotic Bacteria Survival. LWT.

[50] Schülke & Mayr GmbH Product Information: Mikrocount^®^ Duo. https://www.schuelke.com/pl-pl/products.php.

[51] Wu X.J., Wang Y., Yang W., Xie B.H., Yang M.B., Dan W. (2012). A Rheological Study on Temperature Dependent Microstructural Changes of Fumed Silica Gels in Dodecane. Soft Matter.

[52] Patel A.R., Babaahmadi M., Lesaffer A., Dewettinck K. (2015). Rheological Profiling of Organogels Prepared at Critical Gelling Concentrations of Natural Waxes in a Triacylglycerol Solvent. J. Agric. Food Chem..

[53] Łętocha A., Miastkowska M., Sikora E. (2023). Sposób Otrzymywania Mikrokapsułek Zawierających Bakterie Probiotyczne. Patent.

[54] Agudelo J., Cano A., González-Martínez C., Chiralt A. (2017). Disaccharide Incorporation to Improve Survival during Storage of Spray Dried Lactobacillus Rhamnosus in Whey Protein-Maltodextrin Carriers. J. Funct. Foods.

[55] Łętocha A., Miastkowska M., Sikora E. (2022). Probiotic Raw Materials as Active Ingredients of Cosmetic Formulations. Przem. Chem..

[56] Espert M., Hernández M.J., Sanz T., Salvador A. (2022). Rheological Properties of Emulsion Templated Oleogels Based on Xanthan Gum and Different Structuring Agents. Curr. Res. Food Sci..

